# Monitoring Mitochondrial Complex-I Activity Using Novel PET Probe ^18^F-BCPP-EF Allows Early Detection of Radiotherapy Effect in Murine Squamous Cell Carcinoma

**DOI:** 10.1371/journal.pone.0170911

**Published:** 2017-01-26

**Authors:** Chieko Murayama, Akira T. Kawaguchi, Akemi Kamijo, Katsuko Naito, Masakatsu Kanazawa, Hideo Tsukada

**Affiliations:** 1 Department of Clinical Pharmacology, Tokai University School of Medicine, Isehara, Japan; 2 Department of Radiation Oncology, Tokai University School of Medicine, Isehara, Japan; 3 Department of Cell Transplantation and Regenerative Medicine, Tokai University School of Medicine, Isehara, Japan; 4 Support Center for Medical Research and Education, Tokai University, Isehara, Japan; 5 Central Research Laboratory, Hamamatsu Photonics K.K., Hamamatsu, Japan; ENEA Centro Ricerche Casaccia, ITALY

## Abstract

**Objectives:**

Aerobic glycolysis, the main pathway of energy production in tumors (Warburg effect) allows detection of tumors by positron emission tomography (PET) using ^18^F-fluoro-2-deoxy-D-glucose (^18^F-FDG). Since ionizing radiation (IR) is reported to switch aerobic glycolysis to mitochondrial oxidative phosphorylation, radiotherapeutic efficacy was monitored by the activity of mitochondrial complex I (MC-I), using a new PET probe ^18^F-BCPP-EF, ^18^F-2-tert-butyl-4-chloro-5-{6-[2-(2-fluoro-ethoxy)-ethoxy] -pyridine-3-ylmethoxy}-2H-pyridazin-3-one, compared with ^18^F-FDG uptake and the apoptosis index.

**Methods:**

Tumor uptake of ^18^F-BCPP-EF or ^18^F-FDG was examined in C3H/HeN mice inoculated with murine squamous cell carcinoma SCCVII at various time points after a single dose of x-ray irradiation at 0, 6, 15, or 30 Gy. Apoptosis incidence was determined by TUNEL staining in excised tumor tissue.

**Results:**

Tumor growth suppression was dose-dependent; tumor grew 10-fold (0 Gy), 5-fold (6 Gy), 2-fold (15 Gy), and reduced to half in its volume (30 Gy) 14 days after treatment. ^18^F-BCPP-EF uptake was significantly increased as early as 3 days after 15 Gy or 30 Gy, when tumor size and apoptosis index showed no difference among radiation doses. In contrast, ^18^F-FDG uptake was initially increased dose-dependently, remained elevated up to 7 days, and eventually decreased 10 days after 30 Gy and also 14 days after 15 Gy when tumor size was already reduced. Apoptosis index was increased after irradiation but failed to correlate with tumor response.

**Conclusion:**

Tumor uptake of ^18^F-BCPP-EF was increased dose-dependently early after effective doses of IR when ^18^F-FDG uptake as well as apoptosis incidence were not indicative of tumor response. The results suggest that ^18^F-BCPP-EF is a promising “positive” MC-I imaging PET probe for early detection of efficacy of tumor radiotherapy.

## Introduction

While normal cells can generate energy through oxidative phosphorylation in the presence of oxygen (O_2_), this shifts to anaerobic glycolysis as the O_2_ level decreases (the Pasteur effect) [1]. In contrast, malignant tumors can activate glycolysis in the presence of adequate O_2_ (aerobic glycolysis, the Warburg effect) [2], allowing ^18^F-fluoro-2-deoxy -D-glucose (^18^F-FDG) uptake by positron-emission tomography (PET) to be widely used for the detection of tumors [3]. Ionizing radiation (IR) is known to switch tumor aerobic glycolysis to oxidative phosphorylation [4, 5]. Although tumor response to IR may theoretically be monitored by the reduced uptake of ^18^F-FDG, it soon became recognized that increased ^18^F-FDG uptake by IR-induced inflammation interferes with the early detection of tumor response to IR [6]. Therefore, increased activity of oxidative phosphorylation may better be monitored by mitochondrial activity. While the electron transport chain of mitochondria consists of 5 complexes from I to V, complex I (MC-I; reduced nicotinamide adenine dinucleotide-ubiquinone oxidoreductase), is the first, rate-limiting step of intracellular respiratory activity and oxidative phosphorylation. For imaging of MC-I activity, we developed a novel PET probe, ^18^F-2-tert-butyl-4- chloro-5-{6-[2-(2-fluoro-ethoxy)-ethoxy]-pyridin-3-ylmethoxy}-2H-pyridazin-3-one (^18^F-BCPP-EF) and tested it in *in vivo* brains in rat [7, 8] and monkey [9–11]. In the present study, the early detection of tumor radiotherapy was evaluated in murine squamous cell carcinoma SCCVII by comparing the uptake of ^18^F-BCPP-EF with that of ^18^F-FDG as well as the apoptosis incidence.

## Materials and Methods

### Chemicals

Isoflurane was purchased from Sumitomo Dainippon Pharma (Osaka, Japan). Precursor of ^18^F-BCPP-EF and its cold compound were purchased from NARD Institute (Amagasaki, Japan). Mannose triflate and Kryptofix222^®^ (K[2,2,2]) were obtained from ABX (Radeberg, Germany) and Merck (Darmstadt, Germany), respectively.

### PET probe syntheses

Fluorine-18 (^18^F) was produced by ^18^O(p, n)^18^F nuclear reaction using the cyclotron (HM-18, Sumitomo Heavy Industries, Osaka, Japan) at the Hamamatsu Photonics PET center (Hamamatsu, Japan). Labeled compounds were synthesized using a modified CUPID system (Sumitomo Heavy Industries). High performance liquid chromatography (HPLC) analyses of labeled compounds were carried out on a GL-7400 low-pressure gradient HPLC system (GL Sciences, Inc., Tokyo, Japan) with a radioactivity detector (RLC-700, Hitachi Aloka Medical, Inc., Tokyo, Japan).

^18^F-BCPP-EF was radiolabeled by nucleophilic ^18^F-fluorination of its corresponding precursor as reported previously [7]. Radiochemical purity was more than 99%, and specific radioactivity was 139.6 ± 37.0 GBq/μmol.

^18^F-FDG was produced by nucleophilic ^18^F-fluorination of mannose triflate following basic hydrolysis of 2-^18^F-fluoro-1,3,4,6-tetra-*O*-acetyl-D-glucose according to a method described previously [8].

### Animal model

All the animal experiments were reviewed and approved by The Institutional Animal Care and Use Committee at Tokai University (Permit Number: #141012, #152027) and the Central Research Laboratory, Hamamatsu Photonics (Permit Number: HPK-2015-25A and -25B). Female C3H/HeN mice purchased from CLEA Japan, Inc. (Tokyo, Japan) were housed in cages under standard environmental conditions and allowed food and water ad libitum, except the night before PET studies when mice were food deprived. Animals were monitored daily for general and clinical signs of potential adverse effects (eating, drinking, weight change, persistent hypothermia, respiration, weakness, paralysis, incontinence, diarrhea, general behavior). When they showed signs of illness, they were euthanized according to the Guidelines for the Care and Use of Animals for Scientific Purposes at Tokai University and the Central Research Laboratory, Hamamatsu Photonics. Mice inoculated with SCCVII were used for the assessments. SCCVII is a squamous cell carcinoma that arose spontaneously in the abdominal wall of a C3H mouse, was subsequently adapted for clonogenic growth [12], and has been widely used for decades in radiobiological and radiooncological studies [4, 13–15]. SCCVII was inoculated subcutaneously into the right thigh of 8-wk-old mice at a cell density of 1x10^5^ in 0.05 mL of saline [13, 14]. Tumor size was monitored three times a week using caliper throughout the experiment. Tumor volumes were determined using the following formula: tumor volume (Tvol) = *lww*/2, where *l* is length and *w* is width of the tumor. Treatment was started when tumor growth reached 0.19 ± 0.03 cm^3^. All efforts were made to minimize animal suffering. At various time points in 2 weeks after IR, the PET image acquisition was performed under anesthesia with isoflurane. Mice were sacrificed by decapitation under isoflurane anesthesia at either study termination or any of the following clinical endpoints: tumor volume > = 2.0 cm^3^, tumor ulceration, body weight loss > = 20%, or moribund appearance.

### Radiation treatment

Irradiation was delivered in a vertical beam from a 150-kV x-ray unit (MBR-1520R-3, Hitachi Power Solutions Co. Ltd., Hitachi, Japan) and filtered by 0.5 mm Al and 0.2 mm Cu, at room temperature. The mice were restrained in acrylic holders without anesthesia, and local irradiation of tumors was executed at a dose rate of 2.0 Gy/min. The rest of the body was shielded from x-ray by lead plates. The mice received 3 doses of radiation, namely, 6, 15, and 30 Gy.

### Experimental design

The tumor-bearing mice were randomized into 3 groups; group 1 was used for the tumor growth study (8 or 9 mice for each sub-group), group 2 was used for the ^18^F-BCPP-EF uptake and apoptosis index (AI) determination (3 or 4 mice for each time point of the control and irradiated sub-groups), and group 3 was used for the ^18^F-FDG uptake study (3 or 4 mice for each time point of the control and irradiated sub-groups). The tumors were locally exposed to a single radiation dose of 6, 15, or 30 Gy on day 0, and non-irradiated mice served as controls. Tumor size was measured at various time points in group 1, and growth curves were prepared. T_vol_ on day 14 (T_vol_14) after irradiation was determined and used as surrogate of radiotherapeutic effect. The protocol of tumor uptake of the respective PET probes consisted of the sum of two studies. For the first study, we examined tumor uptake of ^18^F-BCPP-EF and ^18^F-FDG at 2 and 7 days after irradiation based on our previous study [14] using the same model of SCCVII, namely, that the reduction of ^18^F-FDG was not observed before 7 days after IR. Since the uptake of ^18^F-FDG on day 7 remained elevated regardless of IR dose, scans for ^18^F-FDG were extended to days 10 and 14 to know its uptake reduction as the second study. Since ^18^F-BCPP-EF uptake showed an obvious dose-dependent increase on day 7, scans for ^18^F-BCPP-EF were broken down to see the early changes between day 2 and day 7 as part of the second study. Consequently, tumor uptake of ^18^F-BCPP-EF and apoptosis detection were examined at 2, 3, 4, 5, and 7 days after irradiation, and uptake of ^18^F-FDG was studied at 2, 7, 10, and 14 days after irradiation.

### PET imaging

PET scans were conducted with a high-resolution small animal PET scanner (Clairvivo- PET, Shimadzu Corporation, Kyoto, Japan) [9]. After overnight fasting, mice were placed in a prone position on a fixation plate under anesthetic condition of 1.5–2.0% isoflurane in O_2_, and then placed in the gantry hole of the PET scanner. After transmission measurement with an external [^137^Cs] point source (22 MBq) for attenuation correction, ^18^F-BCPP-EF or ^18^F-FDG at a dose of ca. 3 MBq was intravenously injected into the tail vein of each mouse. PET data were acquired in list-mode format for 60 min; full 3D sinograms with corrected efficiency, scattering, attenuation, count losses, and decay were reconstructed using an iterative 3D dynamic raw-action maximum likelihood algorithm. Dynamic images as well as summation images of ^18^F-BCPP-EF from 10 to 30 min, and of ^18^F-FDG from 40 to 60 min after injection were reconstructed, creating standard uptake value (SUV) images. Body temperature of mice was monitored and controlled by heating pad during PET measurement. In order to assess the maximum uptake of ^18^F-BCPP-EF or ^18^F-FDG as SUV, regions of interest were placed on PET images of tumor tissues with the aid of individual x-ray CT images obtained just after PET measurement with ClairvivoCT (Shimadzu Corporation).

### Apoptosis detection

At various time points after irradiation, tumor-bearing mice underwent PET scans, were sacrificed, and tumors were excised. Apoptosis induced in tumors was detected by deoxynucleotidyl transferase-mediated deoxyuridine triphosphate-biotin nick end-labeling (TUNEL) method with ApopTag *in situ* apoptosis detection kit (Oncor, Gaithersburg, MD, USA) according to the manufacturer’s instructions. Apoptosis frequency was expressed as the number of positively stained tumor cells among 5000 tumor cells counted by microscopic examination in 4–5 random fields. AI was determined in each tumor as follows: (number of TUNEL-positive tumor cells per section) / (total number of non-necrotic tumor cells per section) x 100.

### Statistical analysis

Statistical analysis was performed with GraphPad Prism for Windows, version 6.0 (GraphPad Software, San Diego, CA, USA). All data were expressed as mean ± SD. Comparisons of T_vol_14, the uptake of each PET ligand and apoptosis frequency between the treatment and control groups were performed by the Mann-Whitney-U test. Correlations between PET ligand uptake and T_vol_14 as well as correlations between apoptosis frequency and T_vol_14 were evaluated by Spearman’s correlation coefficients. P-values < 0.05 were considered statistically significant.

## Results

### Tumor growth

**Fig 1:**
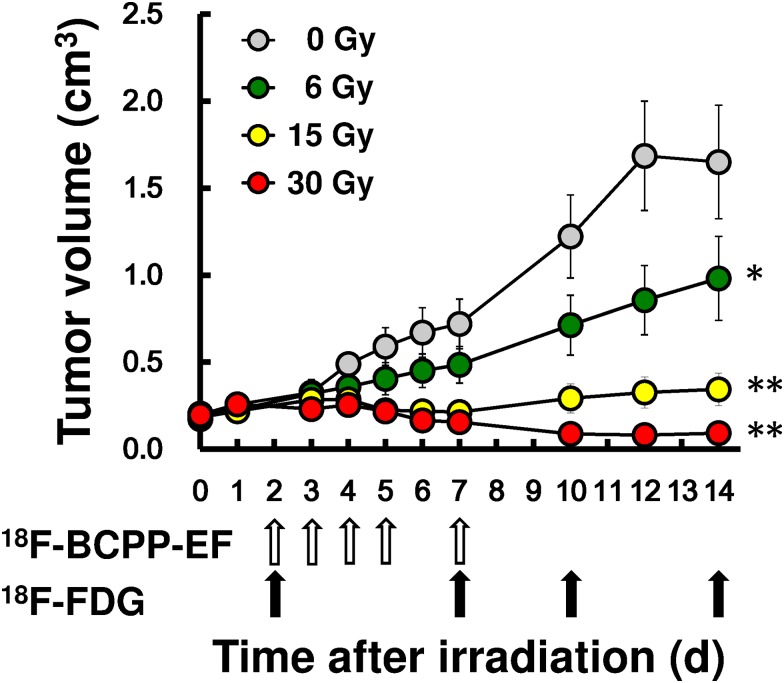
Growth curves of SCCVII tumors after various doses of IR. Tumor growth suppression by IR was shown in terms of tumor volume (T_vol_) and radiation doses; 0 (non-irradiated control), 6, 15, and 30 Gy. PET scan schedule with ^18^F-BCPP-EF (open arrows) and ^18^F-FDG (closed arrows) depicts the timing of respective scans and sacrifice of animals to excise tumors for morphological study. Each point shows mean ± SD for 6 mice. Asterisk(s) indicates significant difference compared with non-treated controls on day 14, * *p*<0.05, ** *p*<0.01.

Tumor growth curves (Fig 1) indicated that tumor size did not differ up to day 3 regardless of IR dose. Non-irradiated tumors in control mice grew to almost 10-fold of original size by day 12 and leveled off thereafter. After irradiation with 6 Gy, tumor growth rate was apparently suppressed, but without shrinkage. Irradiation at 15 Gy resulted in tumor swelling until day 3, followed by continuing shrinkage up to day 7, and then resumption of growth on day 10 and thereafter. No tumor regrowth was observed after irradiation at 30 Gy, which caused progressive shrinkage until day 14. There were significant differences in T_vol_14, when tumor growth was suppressed in reverse order of radiation dose: 6 Gy (*p*<0.05), 15 Gy (*p*<0.01) and 30 Gy (*p*<0.01) compared to non-irradiated control.

### Tumor uptake kinetics of ^18^F-BCPP-EF and ^18^F-FDG

**Fig 2:**
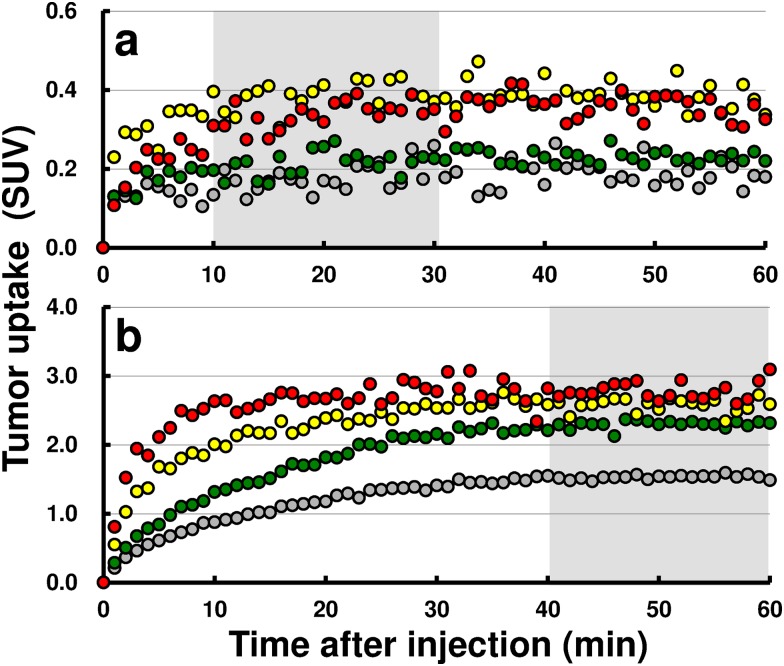
Tumor uptake kinetics of ^18^F-BCPP-EF and ^18^F-FDG. Tumor uptake characteristics of ^18^F-BCPP-EF (a) and ^18^F-FDG (b) were plotted 2 days after IR with 0, 6, 15, and 30 Gy. While uptake reached a plateau earlier for ^18^F-BCPP-EF, allowing SUV uptake calculation between 10 and 30 min, ^18^F-FDG uptake occurred later depending on irradiation dose, necessitating later SUV uptake calculation, between 40 and 60 min after administration. Each point represents mean uptake of 3–4 mice. Gray circle, green circle, yellow circle, red circle represent 0, 6, 15, and 30 Gy, respectively.

Time-activity curves of ^18^F-BCPP-EF (Fig 2a) and ^18^F-FDG (Fig 2b) were plotted to follow the uptake characteristics of the probes in mice 2 days after irradiation with 0, 6, 15, and 30 Gy. While tumor uptake of ^18^F-BCPP-EF reached a plateau earlier (<10 min) regardless of radiation dosage, ^18^F-FDG uptake was slow depending on the radiation dosage, reaching a plateau at 40 min or later. Therefore, uptake was calculated as SUV between 10 and 30 min after injection of ^18^F-BCPP-EF and between 40 and 60 min after injection of ^18^F-FDG, respectively.

### ^18^F-BCPP-EF uptake images

**Fig 3:**
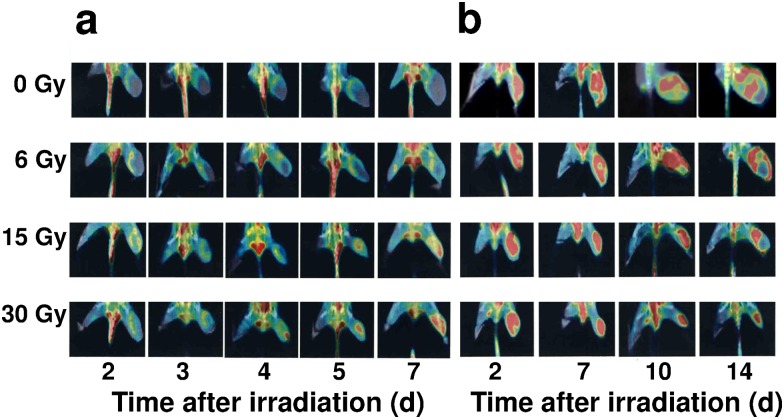
PET/CT fusion images. Representative PET/CT fusion images of ^18^F-BCPP-EF in mice 2, 3, 4, 5, and 7 days after IR with 0 (control), 6, 15, and 30 Gy (a). PET/CT fusion images of ^18^F-FDG were displayed 2, 7, 10 and 14 days after the same IR (b).

PET/CT fusion images of tumors were obtained with ^18^F-BCPP-EF for 7 days (Fig 3a) and ^18^F-FDG for 14 days (Fig 3b) after 0, 6, 15, and 30 Gy of irradiation. In non-irradiated control mice, there was little uptake of ^18^F-BCPP-EF, remaining so until day 7. In mice treated with 6 Gy, ^18^F-BCPP-EF uptake increased gradually with days after irradiation. In contrast, ^18^F-BCPP-EF uptake increased significantly early (2, 3, 4, 5 or 7 days) after irradiation with 15 Gy or 30 Gy, when T_vol_ showed no difference irrespective of radiation dose (Fig 1). In particular, ^18^F-BCPP-EF uptake was significantly increased in parallel to radiation dose on days 3, 4 and 5 after irradiation, when T_vol_ showed little difference (Fig 1). As a result, ^18^F-BCPP-EF uptake images made it possible to identify radiation doses of mice as early as on days 3 through 7 (Fig 3a).

### ^18^F-FDG uptake images

^18^F-FDG uptake in control mice outlined the tumor tissue on all days of scanning except for day 14, when reduced ^18^F-FDG uptake indicated an area of necrosis due to tumor overgrowth (Fig 3b). Tumors in mice treated with 6 Gy showed ^18^F-FDG uptake similar to that of untreated controls. With higher doses, ^18^F-FDG uptake remained elevated until 10 days (30 Gy) and 14 days (15 Gy) after irradiation, when ^18^F-FDG uptake became reduced to be parallel to actual tumor size; radiation dosage could be assumed in the order of ^18^F-FDG uptake on day 2 and in reverse order from day 10 or later.

### Apoptosis

Apoptosis incidence in excised tumor was extremely infrequent in non-irradiated controls, while it was relatively frequent in irradiated tumors regardless of radiation dose (data not shown in Figures).

### Chronological changes in SUV of ^18^F-BCPP-EF, SUV of ^18^F-FDG, and AI

**Fig 4:**
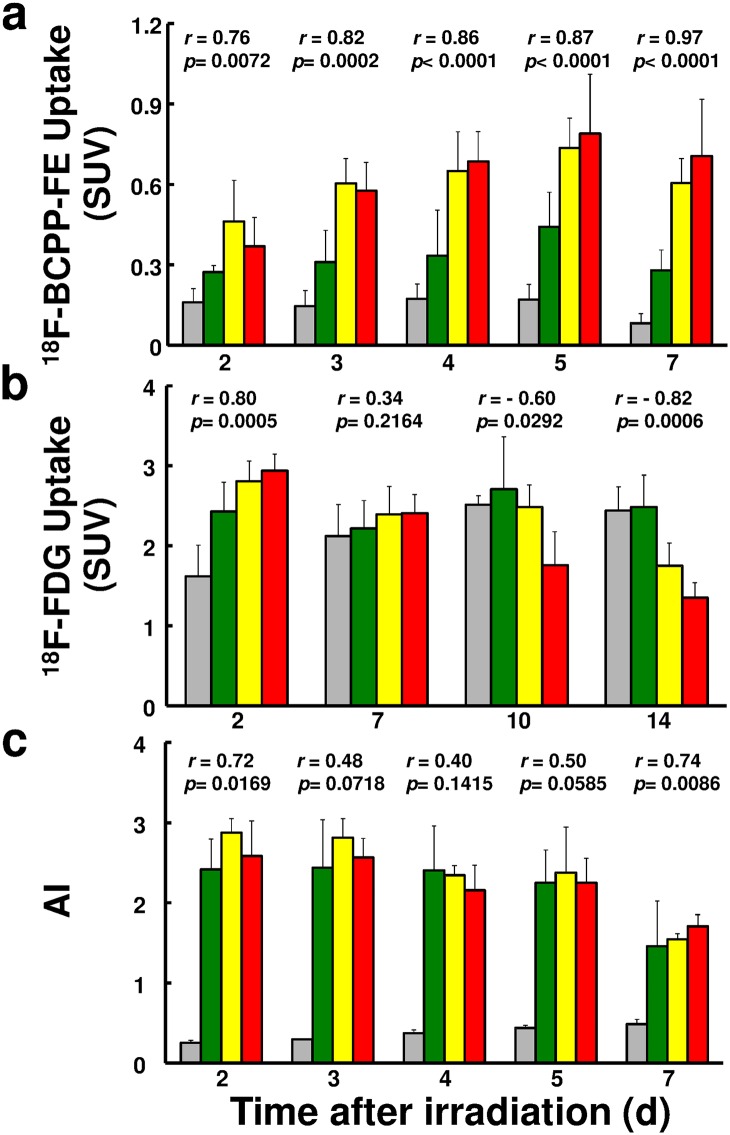
Changes in SUV of ^18^F-BCPP-EF, SUV of ^18^F-FDG and AI compared days after irradiation with 0, 6, 15, and 30 Gy. Changes in uptake of ^18^F-BCPP-EF (a) and ^18^F-FDG (b), and in AI (c) in tumor depicted after irradiation with 0, 6, 15, and 30 Gy. Each column represents mean ± SD for 3–4 mice. Correlation coefficient and significance, respectively, are shown. Gray circle, green circle, yellow circle, red circle represent 0, 6, 15, and 30 Gy, respectively.

Tumor uptakes of ^18^F-BCPP-EF (Fig 4a) and ^18^F-FDG (Fig 4b), as well as AI (Fig 4c), were compared at certain days after irradiation with 0, 6, 15, and 30 Gy. In non-irradiated control mice, the uptakes of ^18^F-BCPP-EF and ^18^F-FDG were relatively constant before day 7 (T_vol_ = 0.72 cm^3^), when ^18^F-BCPP-EF uptake decreased (51.3%) and ^18^F-FDG uptake increased (131.1%) compared to the values on day 2 (T_vol_ = 0.29 cm^3^). In response to radiation, the uptake of ^18^F-BCPP-EF (Fig 4a) increased at all doses starting from day 2 and later, significantly and successively increased compared to non-irradiated control from day 2 with 15 Gy, from day 3 with 30 Gy, and from day 5 with 6 Gy, resulting in a significantly positive correlation between ^18^F-BCPP-EF uptake and radiation doses from day 2 onward, day 3, day 4, day 5, and day 7. As a result, SUV of 0.6 could be a significant cut-off value to identify the therapeutic efficacy as early as 3 days after IR (Fig 4a). In contrast, tumor uptake of ^18^F-FDG (Fig 4b) increased in parallel to the radiation doses on day 2 and remained elevated on day 7 at all doses. SUV of ^18^F-FDG eventually showed a dose-dependent decrease on day 10 (*p* = 0.029) and day 14 (*p* = 0.0006), when tumor size was already reduced (Fig 4b). The time course of AI (Fig 4c) in tumors after irradiation at 6, 15, and 30 Gy revealed significant increases compared to non-irradiated tumors from day 2 onward: day 3, day 4, day 5, and day 7. While elevated AI kept decreasing with time after irradiation regardless of dose, low AI increased consistently in non-irradiated mice along with tumor growth; AI of non-treated tumor doubled between day 2 (0.2%) and day 7 (0.4%), and tumor size (T_vol_) grew 2.62-fold.

### Changes in ^18^F-BCPP-EF, ^18^F-FDG and AI against Tvol on day 14

**Fig 5:**
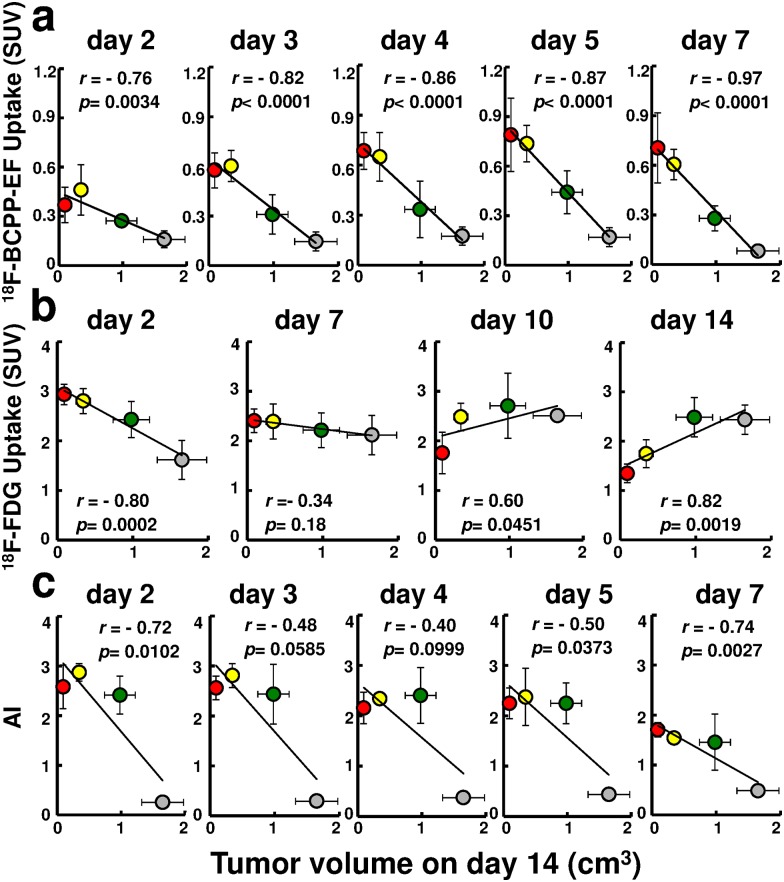
Correlation of uptake of ^18^F-BCPP-EF, uptake of ^18^F-FDG, or AI against T_vol_14 after various doses of IR. Averaged SUV of ^18^F-BCPP-EF (a), ^18^F-FDG (b) and AI (c) in tumor were plotted against the averaged values of T_vol_14 as surrogate of radiotherapeutic effect for mice treated with 0, 6, 15, and 30 Gy. The black line in each scatter-gram represents the correlation for all mice. Correlation coefficient and significance, respectively, are shown. Gray circle, green circle, yellow circle, red circle represent 0, 6, 15, and 30 Gy, respectively.

**Fig 6:**
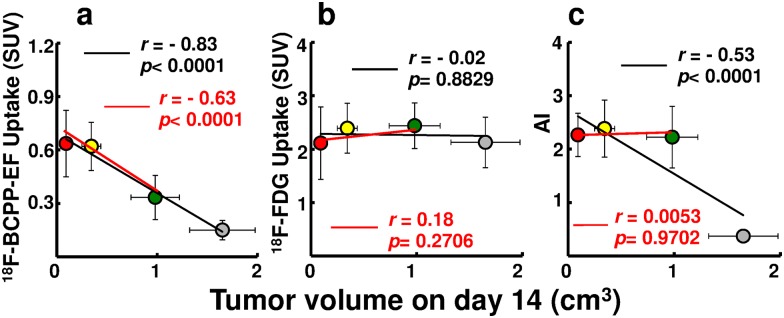
Correlation of uptake of ^18^F-BCPP-EF, uptake of ^18^F-FDG, or AI against T_vol_14 after IR (all days combined). Scatter-plot of each scan (Fig 5) was combined for all radiation doses with SUV of ^18^F-BCPP-EF (a), SUV of ^18^F-FDG (b), and AI (c) listed with T_vol_14. The black line in each scatter-gram represents the correlation for all mice, while the red line represents irradiated tumors without non-treated controls. Correlation coefficient and significance, respectively, are shown. Gray circle, green circle, yellow circle, red circle represent 0, 6, 15, and 30 Gy, respectively.

The uptakes of ^18^F-BCPP-EF (Fig 5a) and ^18^F-FDG (Fig 5b), as well as AI (Fig 5c), were plotted against T_vol_14 as surrogate of radiotherapeutic effect. Highly significant negative correlations were observed between the uptake of ^18^F-BCPP-EF and T_vol_14 as early as on day 2, and on each day up to day 7, and in all days combined (Fig 6a). In contrast, between tumor uptake of ^18^F-FDG and T_vol_14, there was a significant negative correlation on day 2 and positive correlations on days 10 and 14, with no correlation in all days combined (Fig 6b). Since significant negative correlations between AI and T_vol_14 on days 2, 5 and 7 were due mainly to the highly significant difference between irradiated and non-irradiated tumors, there was no significant correlation among irradiated tumors when non-treated mice were excluded (Fig 6c).

## Discussion

Since MC-I is the first, rate-limiting enzyme of the respiratory electron transport chain in mitochondria, ^18^F-BCPP-EF was developed for the quantitative imaging of MC-I activity and tested in rat brain [7, 8] and in primate models of stroke [9], aging [10], and dementia [11]. These studies showed that ^18^F-BCPP-EF specifically marked MC-I activity without being disturbed by inflammation in ischemic brain. Mitochondria, compared to cell nucleus, have been increasingly recognized as a major target of IR, which increases mitochondrial contents or alters mitochondrial functions [5]; a metabolic switching from aerobic glycolysis to oxidative phosphorylation in tumors results in delayed and persistent increase in intracellular reactive oxygen species (ROS) [16–18]. Reversal of this phenomenon was reported by Choi et al [19], who demonstrated that pretreatment with rotenone, a specific MC-I inhibitor, reduces the effect of radiation to induce ROS as well as DNA damage. From these observations, we hypothesized that imaging of tumor mitochondrial bioenergetics might provide a biomarker for monitoring the early response to IR; tumor uptake of ^18^F-FDG is supposed to be reduced due to reversal of the Warburg effect, while ^18^F-BCPP-EF uptake is increased in response to IR. Although tumor shrinkage is an exact marker of anti-tumor therapy, earlier prior to tumor shrinkage is desirable [4, 14] for titration of the therapy—termination due to sufficient response or continuation until desired effect is obtained.

In untreated tumor cells, metabolism under aerobic glycolysis shows markedly low contribution of mitochondria activity, resulting in low uptake of ^18^F-BCPP-EF (Fig 3a). In contrast, IR-induced metabolic switching from aerobic glycolysis to oxidative phosphorylation with high contribution of mitochondria activity induces high ^18^F-BCPP-EF uptake. Such specificity for MC-I caused its uptake to be uninfluenced by inflammation caused by IR in the current study and as observed by microglia in the brain after ischemic damage [9]. The patterns of delayed and protracted uptake of ^18^F-BCPP-EF closely resembled those of mitochondrial ROS production after irradiation in a variety of cell lines [5, 20, 21]. Likewise, the current study disclosed a highly significant positive correlation between ^18^F-BCPP-EF uptake and radiation dose and its efficacy early after IR. In the current study, SUV of 0.6 could be a sensitive and specific cut-off value which identified the therapeutic efficacy as early as 3 days after IR at 15 Gy and 30 Gy, and discriminated inadequate tumor growth suppression in mice treated with 6 Gy that remained under SUV of 0.6 for ^18^F-BCPP-EF (Fig 4a).

While IR-induced inflammation is commonly dose-dependent, its disappearance varies widely, depending on animal species and tissues. The dose-dependent increase in tumor uptake of ^18^F-FDG was apparent on day 2 in the current study and on day 1 as well as day 3 in our previous experiment [14]. ^18^F-FDG uptake remained elevated on day 7, even when it was equivalent to that of untreated tumors regardless of effective IR doses (15 Gy or 30 Gy). These observations are considered to be due to IR-induced inflammation as reported by Chen et al [6] who concluded that ^18^F-FDG PET early after radiotherapy does not accurately reflect the treatment response. In the current study, a slow but consistent increase in ^18^F-FDG uptake and a decrease in ^18^F-BCPP-EF uptake in untreated tumors suggest the persistence of aerobic glycolysis. These observations were in accord with the report by Saito et al [4], who observed a 1.54 fold increase in the lactate/pyruvate ratio during doubled tumor growth. Similarly, in the current study, the 2.56-fold increase in the uptake ratio of ^18^F-FDG/^18^F-BCPP-EF, from 10.1 on day 2 to 25.9 on day 7, was equivalent to the 2.48-fold growth of untreated tumor. These observations suggest that the energy synthesis of untreated tumor shifted even further toward aerobic glycolysis along with tumor growth and that ^18^F-BCPP-EF uptake is comparable to MC-I activity or oxidative phosphorylation. Eventually, ^18^F-FDG uptake decreased at 10 days (30 Gy) to 14 days (15 Gy) after irradiation when macroscopic tumor size was already reduced.

Correlation was highly significant between AI and T_vol_14 in the present study, owing to the fact that untreated tumors were associated with a low AI compared to irradiated tumors with elevated AI regardless of IR dosage. Although AI was low in small untreated tumors, the frequency steadily increased as the tumor grew. For AI to be an index of radiotherapy, IR-induced apoptosis should be significant, dose-dependent, and readily accessible by sampling/biopsy, as reported in cervical cancer where the occurrence of apoptotic cells was associated with better pelvic control [22]. In contrast, the tumor used in the current study was SCCVII, in which IR-induced apoptosis is infrequent and decreases time-dependently, with maximal expression at 6 hours after irradiation [15]. Since apoptosis was determined in tumors excised at least 2 days after IR, AI was not indicative of radiation dose or efficacy in the current study. While EL4 tumor is reported to have AI 10-fold higher than SCCVII after irradiation [15], it shows a therapeutic response equivalent to that of SCCVII. Thus, AI may not necessarily be practical in SCCVII tumor as an index of irradiation effect, as the histological sample, necessarily obtained soon after treatment, would not reflect the treatment response as a whole, being only a microscopic part of the tumor.

While ^18^F-FDG uptake and AI were not indicative of radiation response or efficacy, we speculate that positive imaging of tumor using ^18^F-BCPP-EF was correlated with tumor MC-I activity, ROS production and response to IR [5, 20]. Although we do not have any direct evidence of molecular mechanism(s) regarding how ^18^F-BCPP-EF binds to MC-I after IR, our previous studies confirmed that ^18^F-BCPP-EF and rotenone shared the same binding site called PSST subunit, one of the ubiquinone binding sites of MC-I [8, 10]. Thus, we hypothesize that metabolic switching induces the activation of mitochondrial function, which may accompany the conformational change of protein structure at the PSST binding site from low affinity to high affinity state to facilitate ^18^F-BCPP-EF binding to MC-I. Further studies are required to clarify the mechanism(s). Nonetheless, noninvasive assessment of MC-I function in tumors and/or organs as a whole without requiring tissue sampling will be of great advantage for future clinical applications. While positive imaging of tumor using ^18^F-BCPP-EF may be advantageous in organs with low mitochondrial contents, it would be disadvantageous in tumors in organs with high MC-I activity such as myocardium or kidney [8]. Since it was demonstrated only in SCCVII tumor, additional studies are warranted to examine the behavior of ^18^F-BCPP-EF in various tumors in different organs and timing after anti-tumor treatments.

In conclusion, tumor uptake of ^18^F-BCPP-EF was increased dose-dependently early after effective doses of IR when ^18^F-FDG uptake as well as AI were not indicative of tumor response. The results suggest that ^18^F-BCPP-EF is a promising “positive” MC-I imaging PET probe for early detection of the efficacy of tumor radiotherapy.

## Supporting Information

S1 File This is a raw data for Fig 1. (PDF)

S2 File This is a raw data for Fig 2. (PDF)

S3 File This is a raw data for Fig 4. (PDF)
