# Supplementary material for: Monitoring Mitochondrial Complex-I Activity Using Novel PET Probe 18F-BCPP-EF Allows Early Detection of Radiotherapy Effect in Murine Squamous Cell Carcinoma
Source: PLoS One. 2017 Jan 26;12(1):e0170911. doi: 10.1371/journal.pone.0170911 (PMC5268465; doi:10.1371/journal.pone.0170911)
Supplement: S1 File — (PDF) [file pone.0170911.s001.pdf]

Tumor volume (mm^3)

Day-0				Day-1			
0Gy	6Gy	15Gy	30Gy	0Gy	6Gy	15Gy	30Gy
131.9	195.7	167.5	233.1	184.2	231.1	220.2	279.7
190.2	170.1	154.5	232.4	280.2	204.2	172.7	245.8
223.7	232.4	184.2	188.4	302.0	240.9	220.2	240.2
109.0	220.2	220.2	169.6	254.3	240.4	230.2	273.4
188.4	207.2	190.8	200.2	285.2	245.9	248.6	264.1
195.7	210.2	210.2	198.9	244.9	220.2	223.7	221.9
175.8	188.4	131.9	183.5	282.6	197.8	157.0	290.8
160.1	200.2	179.6	177.9	240.2	200.2	220.2	260.2
179.6	220.2	210.2	195.7	243.7	254.3	273.4	245.8

Day-3				Day-4			
0Gy	6Gy	15Gy	30Gy	0Gy	6Gy	15Gy	30Gy
264.1	395.0	406.2	195.7	446.9	526.6	426.5	220.2
378.5	505.5	328.8	164.9	569.9	545.4	359.8	236.3
323.2	295.8	260.2	240.2	569.1	341.5	245.8	243.0
313.2	241.9	250.2	244.7	466.3	256.1	279.7	247.3
342.4	322.5	279.7	230.2	483.6	360.4	279.7	259.1
329.7	230.2	279.7	221.9	500.8	270.8	264.9	272.0
395.6	248.6	209.3	290.8	490.8	278.0	209.3	282.6
254.3	302.0	260.2	250.2	388.6	318.8	243.7	264.1
329.7	329.7	279.7	236.3	487.5	329.7	264.9	264.9

	Day-5				Day-6		
0Gy	6Gy	15Gy	30Gy	0Gy	6Gy	15Gy	30Gy
583.0	562.7	216.9	237.0	637.8	659.4	201.5	139.2
647.6	513.8	171.7	231.3	755.2	541.7	186.8	154.5
773.9	505.5	260.2	204.6	810.7	489.7	216.7	144.2
620.6	348.6	176.6	185.5	785.8	366.3	197.8	133.9
467.1	379.4	243.7	180.6	498.7	355.5	240.2	113.0
537.1	300.9	243.7	205.1	695.9	377.8	246.1	185.5
680.0	294.4	209.3	196.3	777.2	376.1	191.4	191.8
388.6	388.6	230.2	231.3	357.2	408.0	234.9	196.3
612.3	348.0	264.9	285.2	716.2	484.7	250.2	226.9

Day-7				Day-10			
0Gy	6Gy	15Gy	30Gy	0Gy	6Gy	15Gy	30Gy
663.3	701.6	174.0	131.9	1381.6	900.8	208.8	29.4
857.2	505.5	238.6	144.2	1360.0	744.1	374.2	99.5
833.4	577.8	230.2	119.1	1434.6	1095.1	408.0	105.0
834.1	384.7	158.5	128.2	1220.8	621.7	164.9	59.4
552.6	382.8	266.9	113.0	730.8	602.9	310.9	86.7
706.5	371.6	254.3	194.3	1445.9	571.5	375.0	68.7
857.2	408.2	181.3	167.5	1260.2	549.5	328.1	84.2
423.9	546.4	207.2	184.2	870.8	741.8	188.4	113.1
751.8	486.7	208.8	216.7	1297.9	587.2	273.4	147.2

Day-12				Day-14			
0Gy	6Gy	15Gy	30Gy	0Gy	6Gy	15Gy	30Gy
1933.9	1126.9	233.5	36.0	1801.6	1339.4	222.4	29.4
1892.8	693.9	361.1	101.2	1148.5	796.0	446.5	109.0
1826.3	1233.0	427.4	99.5	1859.4	1389.2	411.6	102.6
2004.1	659.4	176.6	56.1	2130.0	735.9	196.3	71.4
984.5	803.6	338.5	76.5	1154.0	890.2	346.1	95.2
1798.2	796.8	430.3	42.4	1865.2	879.2	413.8	51.0
1578.0				1695.6			
	849.9	247.3	105.0		1064.3	274.8	114.8
1465.3	683.6	391.2	133.9	1548.9	755.2	442.3	166.8
