# Supplementary material for: Monitoring Mitochondrial Complex-I Activity Using Novel PET Probe 18F-BCPP-EF Allows Early Detection of Radiotherapy Effect in Murine Squamous Cell Carcinoma
Source: PLoS One. 2017 Jan 26;12(1):e0170911. doi: 10.1371/journal.pone.0170911 (PMC5268465; doi:10.1371/journal.pone.0170911)
Supplement: S2 File — (PDF) [file pone.0170911.s002.pdf]

Tumo 18F-BCPP-EF Uptake (SUV)

mean					SD				
Time (min)	0Gy	6Gy	15Gy	30Gy	Time (min)	0Gy	6Gy	15Gy	30Gy
0	0.000	0.000	0.000	0.000	0	0.000	0.000	0.000	0.000
1	0.130	0.130	0.230	0.107	1	0.053	0.112	0.018	0.085
2	0.131	0.144	0.292	0.153	2	0.100	0.125	0.048	0.078
3	0.130	0.125	0.287	0.203	3	0.048	0.083	0.050	0.100
4	0.162	0.193	0.308	0.248	4	0.143	0.190	0.073	0.170
5	0.155	0.170	0.246	0.225	5	0.075	0.146	0.060	0.140
6	0.144	0.194	0.345	0.225	6	0.095	0.129	0.065	0.118
7	0.118	0.179	0.348	0.275	7	0.018	0.134	0.027	0.143
8	0.147	0.202	0.348	0.248	8	0.064	0.170	0.090	0.140
9	0.105	0.194	0.333	0.235	9	0.051	0.178	0.074	0.103
10	0.134	0.196	0.395	0.309	10	0.087	0.192	0.071	0.154
11	0.197	0.164	0.344	0.309	11	0.092	0.157	0.099	0.172
12	0.170	0.214	0.330	0.371	12	0.120	0.125	0.060	0.176
13	0.122	0.219	0.386	0.274	13	0.080	0.158	0.076	0.135
14	0.149	0.168	0.397	0.329	14	0.034	0.127	0.084	0.097
15	0.168	0.162	0.410	0.276	15	0.064	0.155	0.121	0.154
16	0.190	0.231	0.304	0.297	16	0.038	0.147	0.057	0.185
17	0.174	0.188	0.390	0.322	17	0.068	0.141	0.029	0.091
18	0.166	0.191	0.371	0.352	18	0.084	0.089	0.073	0.116
19	0.127	0.253	0.395	0.337	19	0.037	0.199	0.125	0.149
20	0.170	0.256	0.412	0.319	20	0.035	0.147	0.025	0.128
21	0.165	0.270	0.367	0.367	21	0.064	0.161	0.029	0.197
22	0.148	0.221	0.369	0.376	22	0.008	0.134	0.036	0.241
23	0.207	0.234	0.428	0.391	23	0.094	0.093	0.047	0.218
24	0.208	0.217	0.423	0.352	24	0.036	0.189	0.065	0.136
25	0.215	0.204	0.366	0.332	25	0.044	0.138	0.039	0.143
26	0.151	0.230	0.425	0.353	26	0.065	0.141	0.036	0.138
27	0.164	0.177	0.433	0.348	27	0.050	0.125	0.116	0.098
28	0.250	0.217	0.387	0.389	28	0.136	0.146	0.042	0.034
29	0.174	0.230	0.383	0.339	29	0.078	0.167	0.043	0.121
30	0.259	0.226	0.370	0.351	30	0.109	0.175	0.046	0.114
31	0.178	0.222	0.378	0.294	31	0.075	0.115	0.085	0.147
32	0.192	0.252	0.356	0.333	32	0.050	0.131	0.052	0.092
33	0.247	0.249	0.434	0.381	33	0.179	0.158	0.106	0.175
34	0.130	0.253	0.472	0.375	34	0.004	0.176	0.146	0.125
35	0.146	0.243	0.374	0.358	35	0.058	0.110	0.054	0.151
36	0.139	0.213	0.386	0.373	36	0.046	0.132	0.017	0.057
37	0.229	0.213	0.384	0.416	37	0.078	0.129	0.042	0.130
38	0.205	0.206	0.388	0.414	38	0.030	0.069	0.005	0.118
39	0.201	0.246	0.362	0.371	39	0.114	0.118	0.039	0.182
40	0.159	0.229	0.442	0.364	40	0.078	0.091	0.078	0.209
41	0.264	0.210	0.372	0.374	41	0.094	0.115	0.025	0.126
42	0.202	0.241	0.397	0.314	42	0.070	0.076	0.062	0.038
43	0.212	0.233	0.379	0.326	43	0.078	0.119	0.058	0.084
44	0.200	0.220	0.385	0.345	44	0.038	0.120	0.052	0.206
45	0.204	0.210	0.390	0.372	45	0.025	0.127	0.037	0.102
46	0.167	0.271	0.428	0.363	46	0.048	0.138	0.044	0.156
47	0.179	0.236	0.392	0.399	47	0.036	0.157	0.022	0.146
48	0.170	0.226	0.375	0.349	48	0.062	0.148	0.033	0.165
49	0.254	0.210	0.381	0.314	49	0.124	0.081	0.021	0.135
50	0.157	0.239	0.359	0.382	50	0.050	0.123	0.049	0.113
51	0.180	0.245	0.386	0.385	51	0.046	0.112	0.044	0.141
52	0.160	0.221	0.448	0.383	52	0.024	0.124	0.050	0.105
53	0.195	0.226	0.334	0.370	53	0.089	0.139	0.026	0.152
54	0.151	0.213	0.381	0.335	54	0.037	0.063	0.053	0.117
55	0.177	0.231	0.411	0.377	55	0.071	0.063	0.077	0.050
56	0.230	0.220	0.339	0.343	56	0.080	0.130	0.033	0.061
57	0.205	0.238	0.352	0.311	57	0.043	0.070	0.045	0.105
58	0.142	0.221	0.413	0.306	58	0.033	0.110	0.044	0.122
59	0.182	0.243	0.375	0.362	59	0.018	0.129	0.044	0.135
60	0.180	0.220	0.337	0.325	60	0.062	0.120	0.040	0.112

Tumo 18F-FDG Uptake (SUV)

mean

Time (min)	0Gy	6Gy	15Gy	30Gy
0	0.000	0.000	0.000	0.000
1	0.208	0.284	0.547	0.805
2	0.364	0.502	1.023	1.520
3	0.461	0.672	1.320	1.941
4	0.548	0.784	1.368	1.842
5	0.608	0.840	1.678	2.110
6	0.672	0.978	1.652	2.242
7	0.727	1.098	1.800	2.491
8	0.772	1.126	1.878	2.425
9	0.863	1.181	1.844	2.521
10	0.873	1.313	2.004	2.630
11	0.909	1.349	1.974	2.641
12	0.933	1.410	2.132	2.470
13	0.990	1.441	2.199	2.522
14	1.017	1.453	2.171	2.568
15	1.016	1.511	2.166	2.659
16	1.103	1.611	2.340	2.755
17	1.120	1.721	2.167	2.747
18	1.140	1.698	2.220	2.629
19	1.162	1.707	2.320	2.673
20	1.175	1.815	2.386	2.668
21	1.262	1.810	2.425	2.731
22	1.291	1.867	2.297	2.595
23	1.229	2.001	2.372	2.675
24	1.339	2.006	2.349	2.880
25	1.344	1.970	2.471	2.586
26	1.366	2.124	2.367	2.675
27	1.370	2.090	2.532	2.941
28	1.386	2.123	2.520	2.901
29	1.335	2.103	2.590	2.814
30	1.408	2.154	2.532	2.771
31	1.391	2.091	2.533	3.056
32	1.497	2.255	2.659	2.811
33	1.448	2.187	2.529	3.072
34	1.459	2.233	2.562	2.703
35	1.436	2.310	2.606	2.653
36	1.449	2.167	2.755	2.949
37	1.512	2.200	2.667	2.809
38	1.478	2.214	2.557	2.632
39	1.538	2.290	2.658	2.336
40	1.547	2.207	2.580	2.815
41	1.516	2.292	2.613	2.698
42	1.479	2.210	2.400	2.753
43	1.514	2.314	2.568	2.737
44	1.468	2.296	2.582	2.741
45	1.524	2.295	2.627	2.825
46	1.524	2.124	2.666	2.880
47	1.529	2.365	2.662	2.879
48	1.564	2.362	2.440	2.925
49	1.495	2.340	2.608	2.706
50	1.540	2.293	2.514	2.630
51	1.532	2.333	2.668	2.709
52	1.549	2.294	2.633	2.937
53	1.528	2.333	2.607	2.695
54	1.542	2.297	2.575	2.668
55	1.534	2.297	2.648	2.737
56	1.590	2.243	2.340	2.825
57	1.530	2.334	2.483	2.593
58	1.570	2.275	2.528	2.659
59	1.531	2.328	2.720	2.924
60	1.484	2.314	2.586	3.090

SD

Time (min)	0Gy	6Gy	15Gy	30Gy
0	0.000	0.000	0.000	0.000
1	0.046	0.092	0.155	0.407
2	0.082	0.125	0.490	0.699
3	0.132	0.128	0.571	0.685
4	0.182	0.143	0.507	0.622
5	0.152	0.140	0.824	0.867
6	0.153	0.225	0.853	0.688
7	0.202	0.224	0.846	0.641
8	0.160	0.182	0.868	0.660
9	0.165	0.239	0.951	0.807
10	0.194	0.276	0.991	0.471
11	0.206	0.252	0.924	0.857
12	0.189	0.294	1.027	0.477
13	0.224	0.352	1.038	0.789
14	0.208	0.264	0.967	0.813
15	0.204	0.360	1.031	0.761
16	0.219	0.427	1.248	0.667
17	0.157	0.502	1.001	0.910
18	0.187	0.384	0.875	0.735
19	0.213	0.356	0.953	0.613
20	0.216	0.359	1.028	0.800
21	0.219	0.397	0.979	0.778
22	0.270	0.363	1.016	0.849
23	0.214	0.464	1.028	0.763
24	0.262	0.491	0.984	0.795
25	0.239	0.429	1.074	0.859
26	0.298	0.371	0.869	0.781
27	0.289	0.362	1.071	1.186
28	0.235	0.498	1.086	0.744
29	0.223	0.447	1.131	1.028
30	0.268	0.382	1.174	1.141
31	0.224	0.455	0.908	1.093
32	0.339	0.464	1.103	1.149
33	0.271	0.380	1.051	1.024
34	0.338	0.499	1.025	0.804
35	0.313	0.435	0.969	0.843
36	0.219	0.454	1.262	1.266
37	0.335	0.434	0.964	0.794
38	0.300	0.421	0.779	1.031
39	0.287	0.477	0.950	0.872
40	0.285	0.367	1.188	1.174
41	0.240	0.563	1.125	1.145
42	0.246	0.426	0.902	0.879
43	0.241	0.511	0.907	1.090
44	0.292	0.508	0.845	0.873
45	0.257	0.449	0.916	0.919
46	0.251	0.386	0.957	1.188
47	0.258	0.458	1.107	1.245
48	0.253	0.487	0.867	1.148
49	0.311	0.428	0.960	1.105
50	0.243	0.428	0.823	0.880
51	0.265	0.472	1.008	1.161
52	0.254	0.336	1.094	1.275
53	0.278	0.332	0.810	1.023
54	0.274	0.450	0.865	1.134
55	0.223	0.467	0.967	1.149
56	0.330	0.515	0.735	0.931
57	0.260	0.423	0.829	1.121
58	0.266	0.391	0.833	1.133
59	0.248	0.439	0.933	1.333
60	0.231	0.510	0.886	1.431
