# Supplementary material for: Monitoring Mitochondrial Complex-I Activity Using Novel PET Probe 18F-BCPP-EF Allows Early Detection of Radiotherapy Effect in Murine Squamous Cell Carcinoma
Source: PLoS One. 2017 Jan 26;12(1):e0170911. doi: 10.1371/journal.pone.0170911 (PMC5268465; doi:10.1371/journal.pone.0170911)
Supplement: S3 File — (PDF) [file pone.0170911.s003.pdf]

TKU_mouse roi SUV
[18F] BCPP-EF

Day-2	Scan_01	Scan_02	Scan_03	Scan_04	mean	SD
0Gy		0.166	0.106	0.207	0.160	0.051
6Gy		0.247	0.298	0.273	0.273	0.025
15Gy		0.314	0.621	0.450	0.462	0.154
30Gy		0.261	0.477	0.371	0.369	0.108

Day-3	Scan_01	Scan_02	Scan_03	Scan_04	mean	SD
0Gy	0.158	0.071	0.209	0.148	0.147	0.057
6Gy	0.147	0.421	0.304	0.368	0.310	0.119
15Gy	0.474	0.675	0.592	0.671	0.603	0.094
30Gy	0.428	0.633	0.670	0.575	0.577	0.106

Day-4	Scan_01	Scan_02	Scan_03	Scan_04	mean	SD
0Gy	0.207	0.136	0.120	0.233	0.174	0.055
6Gy	0.413	0.232	0.160	0.532	0.334	0.170
15Gy	0.648	0.817	0.461	0.674	0.650	0.146
30Gy	0.675	0.552	0.698	0.821	0.686	0.110

Day-5	Scan_01	Scan_02	Scan_03	Scan_04	mean	SD
0Gy	0.114	0.172	0.248	0.147	0.170	0.057
6Gy	0.547	0.311	0.560	0.349	0.442	0.130
15Gy	0.631	0.666	0.876	0.773	0.736	0.111
30Gy	0.721	0.584	0.748	1.104	0.789	0.222

Day-7	Scan_01	Scan_02	Scan_03	Scan_04	mean	SD
0Gy		0.068	0.109	0.086	0.088	0.021
6Gy		0.349	0.198	0.292	0.280	0.076
15Gy		0.597	0.639	0.582	0.606	0.030
30Gy		0.699	0.726	0.708	0.711	0.013

TKU_mouse roi SUV
[18F] FDG

Day-2	Scan_01	Scan_02	Scan_03	Scan_04	mean	SD
0Gy	1.140	2.099	1.621	1.610	1.618	0.392
6Gy	2.370	2.338	2.074	2.940	2.430	0.365
15Gy	2.628	3.165	2.804	2.624	2.805	0.254
30Gy	2.922	3.154	2.744		2.940	0.206

Day-7	Scan_01	Scan_02	Scan_03	Scan_04	mean	SD
0Gy	1.652	2.007	2.228	2.596	2.121	0.396
6Gy	1.964	2.724	2.009	2.167	2.216	0.350
15Gy	2.690	2.088	2.704	2.086	2.392	0.352
30Gy	2.147	2.608	2.463		2.406	0.236

Day-10	Scan_01	Scan_02	Scan_03	Scan_04	mean	SD
0Gy	2.402	2.632	2.497		2.510	0.116
6Gy	2.352	3.465	2.306		2.708	0.656
15Gy	2.179	2.719	2.557		2.485	0.277
30Gy	2.097	1.291	1.884		1.757	0.418

Day-14	Scan_01	Scan_02	Scan_03	Scan_04	mean	SD
0Gy	2.121	2.477	2.716		2.438	0.299
6Gy	2.182	2.938	2.336		2.486	0.399
15Gy	1.838	1.434	1.979		1.750	0.283
30Gy	1.458	1.464	1.133		1.352	0.189

AI

mouse No	Day	Dose(Gy)	Tissue_01	Tissue_02	Tissue_03	Tissue_04	Ave	mean	SD
125	2	0	0.3	0.25	0.2	0.25	0.25		
128	2	0	0.15	0.25	0.3	0.2	0.23		
180	2	0	0.25	0.3	0.3	0.3	0.29	0.25	0.03
124	2	6	3	2.5	2.5	2	2.5		
196	2	6	2	2	2	2	2.0		
134	2	6	3	2.5	3	2.5	2.8	2.42	0.4
126	2	15	2.5	3	3	2.5	2.8		
136	2	15	3	3	3	3	3.0		
166	2	15	0	1	0	0		2.88	0.2
162	2	30	2.5	2.5	3	2.5	2.6		
163	2	30	2	2	2	2.5	2.1		
176	2	30	3	3	3	3	3.0	2.58	0.4
413	3	0	0.25	0.3	0.3	0.35	0.3		
432	3	0	0.35	0.3	0.25	0.3	0.3		
450	3	0	0.25	0.3	0.3	0.3	0.3	0.30	0.01
235	3	6	2.5	3	3	2.5	2.8		
403	3	6	2.5	2.5	2	2.5	2.4		
406	3	6	1.5	2	1.5	1.5	1.6		
423	3	6	3	3	3	3	3.0	2.44	0.6
234	3	15	2.5	2.5	2.5	2.5	2.5		
433	3	15	3	3	3	3	3.0		
434	3	15	3	2.5	2.5	3	2.8		
443	3	15	3	3	3	3	3.0	2.81	0.2
411	3	30	2.5	2.5	2.5	2	2.4		
445	3	30	3	3	3	2.5	2.9		
451	3	30	2.5	3	2.5	2.5	2.6		
468	3	30	2	2.5	2.5	2.5	2.4	2.56	0.2
408	4	0	0.45	0.45	0.35	0.4	0.4		
412	4	0	0.4	0.35	0.35	0.4	0.4		
436	4	0	0.25	0.4	0.35	0.35	0.3	0.38	0.0
407	4	6	2	1.5	2.5	2	2.0		
419	4	6	3	3	3	3	3.0		
453	4	6	3	2.5	2.5	3	2.8		
465	4	6	1.5	2	2	2	1.9	2.41	0.6
430	4	15	2.5	2.5	2	2.5	2.4		
459	4	15	1.5	2.5	2.5	2.5	2.3		
462	4	15	2.5	2	2	2.5	2.3		
471	4	15	2	2.5	3	2.5	2.5	2.34	0.1
405	4	30	2.5	3	2.5	2.5	2.6		
435	4	30	2	2	2	2	2.0		
449	4	30	2	2	2	2	2.0		
470	4	30	2	2	2	2	2.0	2.16	0.3
402	5	0	0.5	0.45	0.45	0.5	0.5		
414	5	0	0.35	0.45	0.4	0.45	0.4		
440	5	0	0.45	0.5	0.4	0.35	0.4	0.44	0.0
437	5	6	2	2.5	2.5	2	2.3		
448	5	6	2.5	2.5	3	3	2.8		
454	5	6	1.5	2	1.5	2	1.8		
483	5	6	2	2.5	2.5	2	2.3	2.25	0.4
401	5	15	2	2.5	2.5	2.5	2.4		
415	5	15	1.5	1.5	1.5	2	1.6		
416	5	15	2.5	2.5	2.5	2.5	2.5		
421	5	15	3	3	3	3	3.0	2.38	0.6
236	5	30	1.5	2	2	2	1.9		
426	5	30	2	2.5	2	2.5	2.3		
439	5	30	2	2	2.5	2.5	2.3		
469	5	30	2.5	2.5	2.5	3	2.6	2.25	0.3
157	7	0	0.6	0.45	0.4	0.55	0.5		
168	7	0	0.45	0.4	0.35	0.5	0.4		
194	7	0	0.6	0.55	0.45	0.55	0.5	0.49	0.1
137	7	6	2	2	2	2	2.0		
154	7	6	1.5	1.5	1.5	1.5	1.5		
199	7	6	1	0.5	1.5	0.5	0.9	1.46	0.6
161	7	15	1.5	1.5	1.5	1.5	1.5		
171	7	15	2	1.5	1.5	1.5	1.6		
172	7	15	1.5	1.5	1.5	1.5	1.5	1.54	0.1
156	7	30	2	1.5	1.5	1.5	1.6		
170	7	30	1.5	2	2	2	1.9		
177	7	30	2	1.5	1.5	1.5	1.6	1.71	0.1
